# A Vision Transformer Approach for Fully Automated and Scalable Dementia Screening using Clock Drawing Test Images

**DOI:** 10.1002/alz70858_097670

**Published:** 2025-12-24

**Authors:** Michael B Bone, Morris Freedman, Sandra E. Black, Daniel Felsky, Sanjeev Kumar, Bradley Pugh, Stephen C Strother, David F. Tang‐Wai, Carmela Tartaglia, Bradley R Buchsbaum

**Affiliations:** ^1^ Rotman Research Institute at Baycrest, Toronto, ON, Canada; ^2^ University of Toronto, Toronto, ON, Canada; ^3^ Toronto Dementia Research Alliance, Toronto, ON, Canada; ^4^ Baycrest Health Sciences, Toronto, ON, Canada; ^5^ Mt. Sinai Hospital, Toronto, ON, Canada; ^6^ Sunnybrook Research Institute, Toronto, ON, Canada; ^7^ Centre for Addiction and Mental Health, Toronto, ON, Canada; ^8^ Department of Psychiatry, University of Toronto, Toronto, ON, Canada; ^9^ Department of Medicine, University of Toronto, Toronto, ON, Canada; ^10^ Memory Clinic, Toronto Western Hospital, University Health Network, Toronto, ON, Canada; ^11^ Tanz Centre for Research in Neurodegenerative Diseases, University of Toronto, Toronto, ON, Canada; ^12^ Toronto Dementia Research Alliance (TDRA), Toronto, ON, Canada

## Abstract

**Background:**

The clock drawing test (CDT) has been used as a cognitive screening tool for Alzheimer's disease (AD) and other forms of dementia. However, its clinical utility is constrained by requirements for trained scorers and non‐standardized diagnostic criteria.

**Method:**

We developed a fully‐automated vision transformer (ViT)‐based dementia diagnostic pipeline incorporating convolutional neural network (CNN) preprocessing of hand drawn CDT images. The architecture implements fine‐tuned ViT feature extraction followed by linear classification for dementia prediction. The method was trained using the National Health and Aging Trends Study (NHATS) dataset (*n* = 54027) and tested on an independent clinical cohort from the Toronto Dementia Research Alliance (TDRA) (*n*(dementia diagnosis) = 522, *n*(normal cognition) = 340).

**Results:**

The ViT‐based approach demonstrated superior predictive performance (balanced accuracy = 76.5%) compared to both traditional human‐scored CDT features (balanced accuracy = 74.3%) and three published deep learning architectures when evaluated on the TDRA dataset (balanced accuracy: MiniVGG = 73.3%, MNv2 = 72.3%, RF‐VAE = 69.1%).

**Conclusion:**

This pen‐and‐paper compatible, transformer‐based diagnostic system enables scalable remote cognitive screening through automated CDT image analysis that is competitive with human‐scored features, potentially increasing diagnostic accessibility and comfort for elderly populations across diverse socioeconomic contexts.

